# Characterization of a novel model of global forebrain ischaemia–reperfusion injury in mice and comparison with focal ischaemic and haemorrhagic stroke

**DOI:** 10.1038/s41598-020-75034-4

**Published:** 2020-10-23

**Authors:** Natasha Ting Lee, Carly Selan, Joanne S. J. Chia, Sharelle A. Sturgeon, David K. Wright, Akram Zamani, Melrine Pereira, Harshal H. Nandurkar, Maithili Sashindranath

**Affiliations:** 1 Australian Centre for Blood Diseases, Central Clinical School, Alfred Hospital, Monash University, Melbourne, VIC 3004 Australia; 2 Department of Neuroscience, Central Clinical School, Monash University, Melbourne, VIC 3004 Australia; 3 Present Address: Australian Centre for Blood Diseases, Central Clinical School, Alfred Hospital, Monash University, Monash AMREP Building, Level 1, Walkway, via The Alfred Centre, 99 Commercial Road, Melbourne, VIC 3004 Australia

**Keywords:** Stroke, Cerebrovascular disorders, Experimental models of disease

## Abstract

Stroke is caused by obstructed blood flow (ischaemia) or unrestricted bleeding in the brain (haemorrhage). Global brain ischaemia occurs after restricted cerebral blood flow e.g. during cardiac arrest. Following ischaemic injury, restoration of blood flow causes ischaemia–reperfusion (I/R) injury which worsens outcome. Secondary injury mechanisms after any stroke are similar, and encompass inflammation, endothelial dysfunction, blood–brain barrier (BBB) damage and apoptosis. We developed a new model of transient global forebrain I/R injury (dual carotid artery ligation; DCAL) and compared the manifestations of this injury with those in a conventional I/R injury model (middle-cerebral artery occlusion; MCAo) and with intracerebral haemorrhage (ICH; collagenase model). MRI revealed that DCAL produced smaller bilateral lesions predominantly localised to the striatum, whereas MCAo produced larger focal corticostriatal lesions. After global forebrain ischaemia mice had worse overall neurological scores, although quantitative locomotor assessment showed MCAo and ICH had significantly worsened mobility. BBB breakdown was highest in the DCAL model while apoptotic activity was highest after ICH. VCAM-1 upregulation was specific to ischaemic models only. Differential transcriptional upregulation of pro-inflammatory chemokines and cytokines and TLRs was seen in the three models. Our findings offer a unique insight into the similarities and differences in how biological processes are regulated after different types of stroke. They also establish a platform for analysis of therapies such as endothelial protective and anti-inflammatory agents that can be applied to all types of stroke.

## Introduction

There are three major pathological types of stroke– ~ 80% of strokes are ischaemic, and the remainder are haemorrhagic (~ 15% are caused by intracerebral haemorrhage (ICH) and ~ 5% by sub-arachnoid haemorrhage)^1^. Global ischaemic brain injury occurs after cardiac arrest or prolonged hypotensive episodes following surgery/trauma. Significant neurological deficits result not only due to brain ischaemia sustained during cardiac arrest itself but also due to reperfusion injury, which involves impaired cerebral autoregulation, hypoperfusion, and oedema in the hours to days after return of spontaneous circulation. It is the primary cause of death in 68% of inpatient and 23% of out-of-hospital cardiac arrest cases^2^. Approximately 50% of ischaemic strokes occur due to thrombi and/or emboli triggering transient or permanent reduction in cerebral blood flow in a major brain artery. Primary injury in ICH results from blood vessel rupture leading to extravasation of blood components into the brain^1^. Even though ischaemic and haemorrhagic strokes manifest differently but both impact long-term cerebral and functional recovery.

The primary injury after stroke constitutes neuronal damage due to energy deprivation and ATP consumption, disrupting ionic imbalance and prompting glutamate release and subsequent excitotoxicity and oxidative stress. These altered hemodynamic and molecular responses result in secondary brain injury^3^. In ICH, thrombin activation and release of clot components (iron and haemoglobin), as well as direct mechanical damage caused by the haematoma mass effect further promote secondary injury. Increased intracranial pressure and/or subsequent pro-thrombotic/pro-inflammatory processes result in formation of ischaemic lesions distal to the initial haematoma^4^. Regardless of the type of stroke, these processes culminate in endothelial activation characterised by upregulation of vascular cell adhesion molecule (VCAM)-1, as well as neuronal death/neurological dysfunction, blood–brain barrier (BBB) disruption, and consequent cerebral oedema. Furthermore after ischaemia, sudden restoration of blood flow causes ischaemia/reperfusion (I/R) injury which further aggravates inflammation, oxidative stress and cell death and can result in haemorrhagic transformation^5^.

Majority of pre-clinical studies in neuroprotection have failed to translate into clinical management of stroke. The Stroke Therapy Academic Industry Roundtable (STAIR) stipulates that animal studies be conducted in > 1 stroke model and also recommend that a range of structural and functional readouts, including behavioural tests to assess neurological function be used for evaluating drug efficacy^6^.

Comparing effects of potential therapies in ischaemic as well as haemorrhagic stroke is rarely performed in the laboratory setting. This is particularly important for addressing cytoprotection to treat stroke e.g. facilitating endothelial protection as a means to salvage the ischaemic penumbra and reduce secondary ICH. As a corollary, in primary ICH cases, protecting endothelial cells, astrocytes and pericytes might reduce haematoma expansion and oedema. Characterising BBB breakdown and oedema as well as bleeding in the brain is further valid during development of new antiplatelet, anticoagulant, or fibrinolytic therapies for ischaemic stroke, to test haemorrhagic transformation of an ischaemia-induced infarct^7^.

We developed a new model of transient global forebrain ischaemia (Dual Carotid Artery Ligation; DCAL) to study the neurological effects of cardiac arrest. We compared cell death and neurological outcomes as well as inflammation and BBB damage in this model to the middle cerebral artery occlusion (MCAo) model of transient focal ischaemia and the collagenase ICH model at 24 h post-stroke.

## Results

The DCAL model is a reproducible and reliable method to induce global forebrain ischaemia using a simple and quick surgical method. To assess/confirm the extent of cessation and subsequent reperfusion of blood flow during the procedure, we i.v. injected Evans blue into the mice post bilateral carotid artery ligation as well as DCAL (transient occlusion of the right CCA) to determine the extent of ischaemia and reperfusion in this model. Following bilateral carotid artery ligation, Evan’s blue is excluded from the vasculature of the brain spanning the frontal and parietotemporal lobes (Bregma 2 to − 4 mm) but not the cerebellum. This was uniform in both the left and right sides of the brain (DCAL- non-perfused) (Figure S1A and B). When the regular DCAL procedure was performed i.e. the left carotid artery was clamped for 30 min and then allowed to reperfuse (DCAL-reperfused), significantly higher Evan’s blue signal was detected in both the left (Figure S1A) and right hemispheres (Figure S1B), suggesting strong reperfusion and therefore subsequent reperfusion injury. In sham and naïve animals, similar levels of Evan’s blue was detected in both sides of the brain however, only the naïve group had significantly higher levels of Evan’s blue compared to the non-perfused brain for the mid-section (0–2 mm anterior to bregma) of the left side of the brain (Figure S1A) and the latter section (2–4 mm posterior to bregma) of the right side of the brain (Figure S1B). This suggests that there could be lasting perfusion impairment following DCAL and sham procedures. This further explains why the mice subjected to the sham procedure have a degree of cerebral damage.

### Neurological deficits are more pronounced while locomotor function is preserved in mice that have undergone DCAL compared to MCAo and ICH

**Figure 1:**
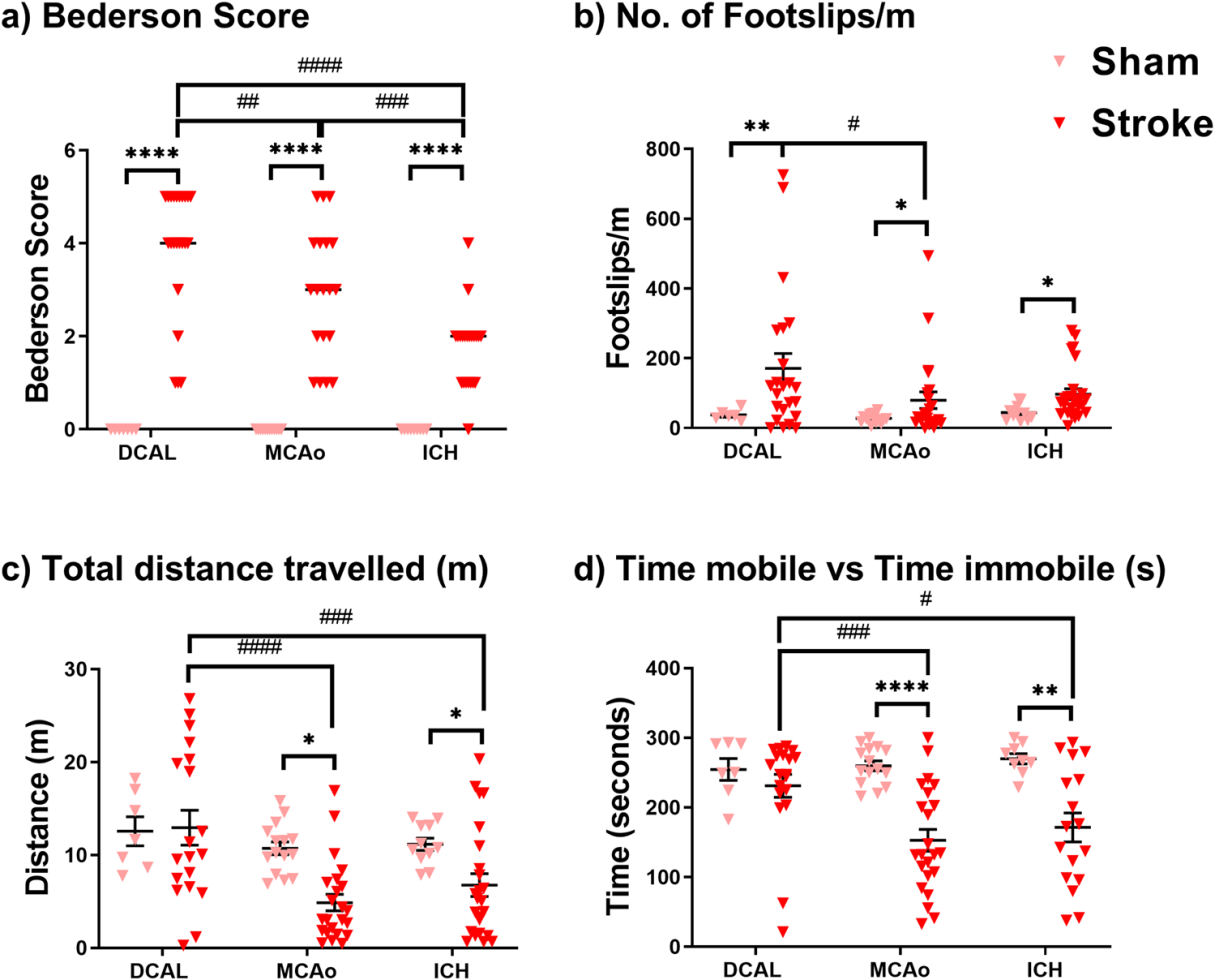
Neurological and locomotor tests identify significant changes in neurobehavioural outcome in all three stroke models. (**a**) DCAL, MCAo and ICH mice had an overall higher Bederson score compared to respective sham controls (Sham: n = 7–15, Stroke: n = 21–23; Bars indicate median value, ****p < 0.00001, stroke vs sham. ##p < 0.001, ###p < 0.0001, ####p < 0.00001; DCAL vs MCAo vs ICH; two‐way analysis of variance (ANOVA)). There were pronounced locomotor and neurological deficits in all the stroke mice based on (**b**) Number of Footslips/m and (**c**) Total distance travelled (m) only significantly decreased after focal stroke (MCAo and ICH) (Sham: n = 7–15, Stroke: n = 19–23; unpaired t-test, two-way ANOVA with Tukeys post-hoc analysis). (**d**) Total duration of mobile episodes (s) was also measured using the open field assay and time mobile was again found to be only significantly decreased after focal stroke (MCAo and ICH) (Data is Mean ± SEM, Sham: n = 7–15, Stroke: n = 19–23; *p < 0.05, **p < 0.001, ****p < 0.00001; unpaired t-test, sham vs stroke, #p < 0.05, ###p < 0.0001, ####p < 0.00001, two‐way ANOVA with Tukeys post‐hoc analysis, DCAL vs MCAo vs ICH).

As expected after DCAL, MCAo and ICH procedures mice had an overall higher median Bederson score compared to sham controls (Fig. 1a). However, compared to ICH and MCAo, DCAL stroke caused the highest median Bederson score among the models.

MCAo resulted in two-fold increase in footslips/m (Fig. 1b) but deficits were significantly more pronounced after DCAL (Fig. 1b). There were also significant increases in footslips/m after ICH. In the open field assay, mice travelled 50% less distance after MCAo and ICH compared to respective sham animals (Fig. 1c) and also travelled significantly less compared to mice after DCAL. Interestingly, despite other evident neurological deficits, after DCAL, distance travelled and time mobile was not reduced compared to sham animals (Fig. 1c, d. Mice subjected to MCAo and ICH were substantially less mobile compared to sham animals (Fig. 1d). Further, these mice were also significantly less active when compared to mice after DCAL (Fig. 1d).

### DCAL produces smaller bilateral lesions relative to MCAo but not ICH

**Figure 2:**
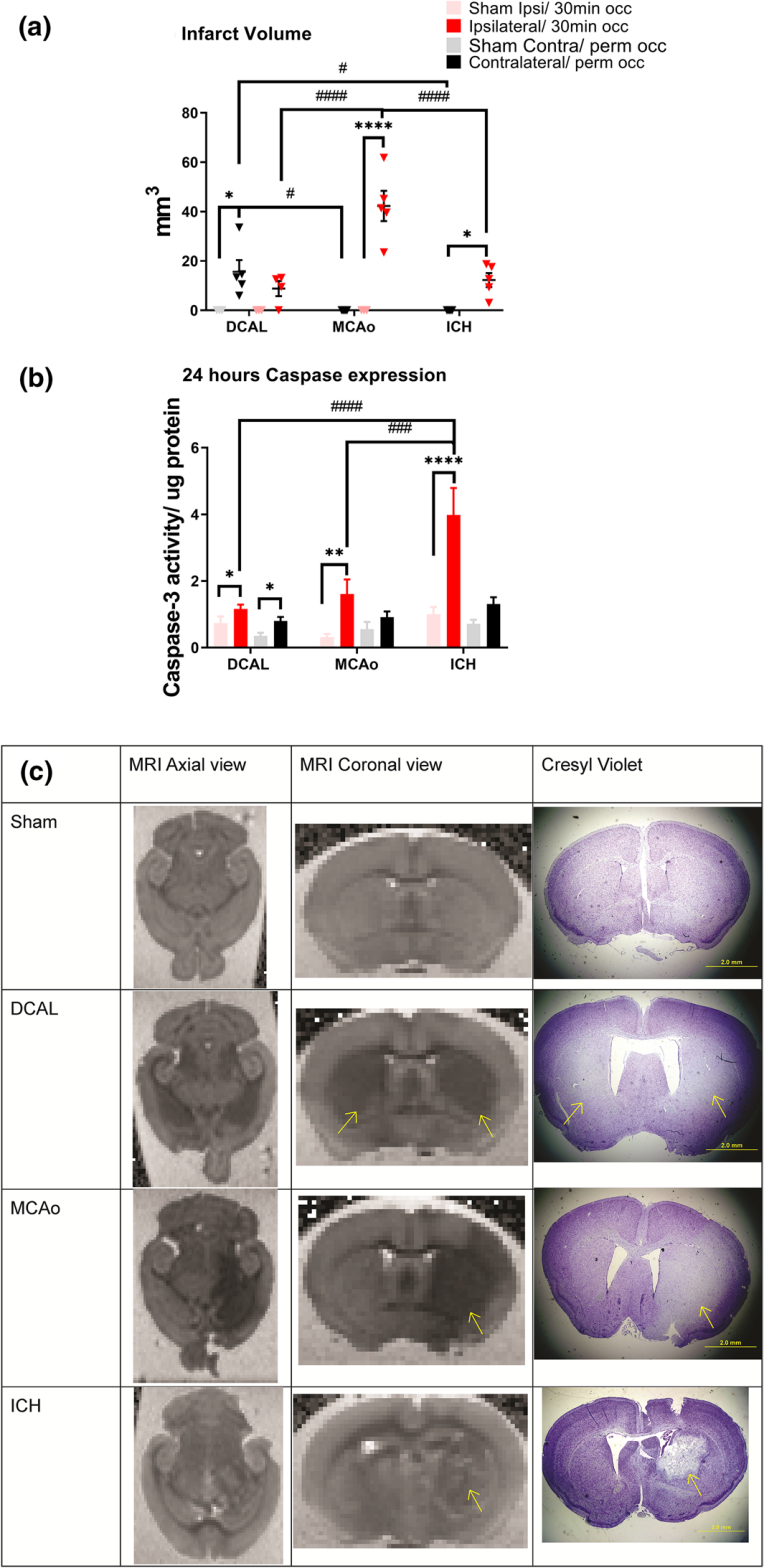
Infarct volume and apoptotic activity are clearly quantifiable 24 h post-stroke in all three models (**a**) MRI-based DWI-quantified infarct volumes (mm^3^) of mouse brains 24 h post-stroke are all significantly higher when compared to its respective sham, but only DCAL and MCAo have significant lesions in the site of injury as opposed to ICH. The MCAo model consistently produces larger infarct sizes as compared to the DCAL and ICH model. (Data is Mean ± SEM; Sham: n = 3–5, Stroke: n = 5; *p < 0.05, ****p < 0.00001; one‐way ANOVA with Sidak post‐hoc analysis, sham vs stroke, #p < 0.05, ####p < 0.00001, two-way ANOVA with Tukeys post‐hoc analysis, DCAL vs MCAo vs ICH). (**b**) Caspase activity 24-h post procedure is significantly increased in the site of injury in the DCAL, MCAo and ICH models. As expected, significant apoptotic activity is seen in both hemispheres in only the DCAL model. (Data is Mean ± SEM; Sham: n = 4–5, Stroke: n = 6–10; ****p < 0.00001; one‐way ANOVA with Uncorrected Fishers LSD test, sham vs stroke. ###p < 0.0001, ####p < 0.00001, two‐way ANOVA with Tukeys post‐hoc analysis, DCAL vs MCAo vs ICH). (**c**) MR imaging and Cresyl Violet staining of mouse brains after sham, DCAL, MCAo and ICH procedures (n = 3–5) distinctly show the infarcted area indicated with the arrow, and this injury and corresponding absence of Cresyl violet positive cells in the same region, confirming cell death.

Infarcts were visible in both hemispheres in the DCAL model (Fig. 2a), however only the left hemisphere (site of permanent occlusion) had a significantly larger lesion compared to the sham animals (mean volume = 15.6 mm^3^; Fig. 2a). As expected, MCAo stroke resulted in a unilateral infarct in the right hemisphere (mean = 42.3 mm^3^; Fig. 2). When compared, MCAo yielded significantly larger lesion volume than the lesion volume caused by DCAL-stroke (n = 5; p = 0.0002). Despite comparable neurological deficits, the mean infarct volume after ICH was less than ischaemic stroke (mean = 12.3 mm^3^; Fig. 2) but was similar to infarct volume in the DCAL model. Corresponding cresyl violet stained sections taken from the same brains clearly delineated the infarcts in all three stroke models (Fig. 2c).

### Apoptotic activity is comparable between DCAL and MCAo models but higher after ICH

Both ischaemia and haemorrhage triggered apoptotic cell death in the brain at 24 h (Fig. 2b). After DCAL-stroke, caspase 3/7 activity was significantly higher in both sides of the brain (Fig. 2b). After ICH, caspase activity increased 2 × in the haemorrhaged site, and notably, this model resulted in the highest apoptotic activity amongst the 3 models (Fig. 2b) despite resulting the lowest lesion volume.

### Albumin extravasation is increased at 24 h post injury after DCAL, MCAo and ICH

**Figure 3:**
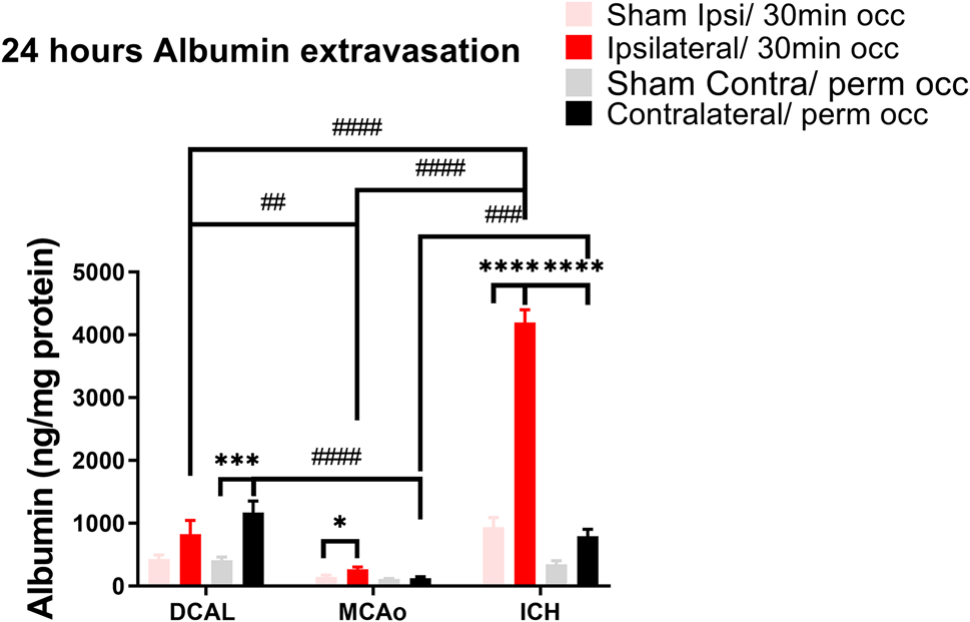
BBB permeability measured by albumin extravasation in the brain parenchyma is significantly increased within 24 h post injury, it is further increased in DCAL, MCAo and ICH mice. (Data is Mean ± SEM; Sham: n = 5, Stroke: n = 8; *p < 0.005, ***p < 0.0001, ****p < 0.00001, sham vs stroke, ##p < 0.001, ###p < 0.0001, ####p < 0.00001; two‐way ANOVA with Tukey post‐hoc analysis, DCAL vs MCAo vs ICH).

At 24 h post-DCAL, BBB permeability increased in the site of transient occlusion, but there was a further two-fold increase in the side of permanent occlusion, consistent with increased lesion volume in this side of the brain (Fig. 3). BBB permeability was significantly increased after MCAo, albeit significantly less than the DCAL model (Fig. 3). Albumin extravasation was highest overall after ICH (Fig. 3). However, there is an obvious caveat in that albumin would increase if there was bleeding in the brain. Therefore, we measured and correlated albumin and haemoglobin levels in the peri-haematomal region. Haemoglobin was increased both at 3 and 24 h post-ICH (Figure S2). At 3 h, albumin and haemoglobin levels correlated, confirming that the high amount of albumin was in part due to the accumulation of blood (n = 8; Pearson r = 0.68; p = 0.0116-not shown). However, at 24 h, there was no correlation between albumin and haemoglobin levels (n = 8; Pearson r = 0.27; P = 0.55-not shown), suggesting that albumin content detected within the brain parenchyma at this time was due to plasma protein extravasation as a result of increased BBB permeability.

### VCAM expression varies at 3 h and 24 h post injury after DCAL, MCAo and ICH

**Figure 4:**
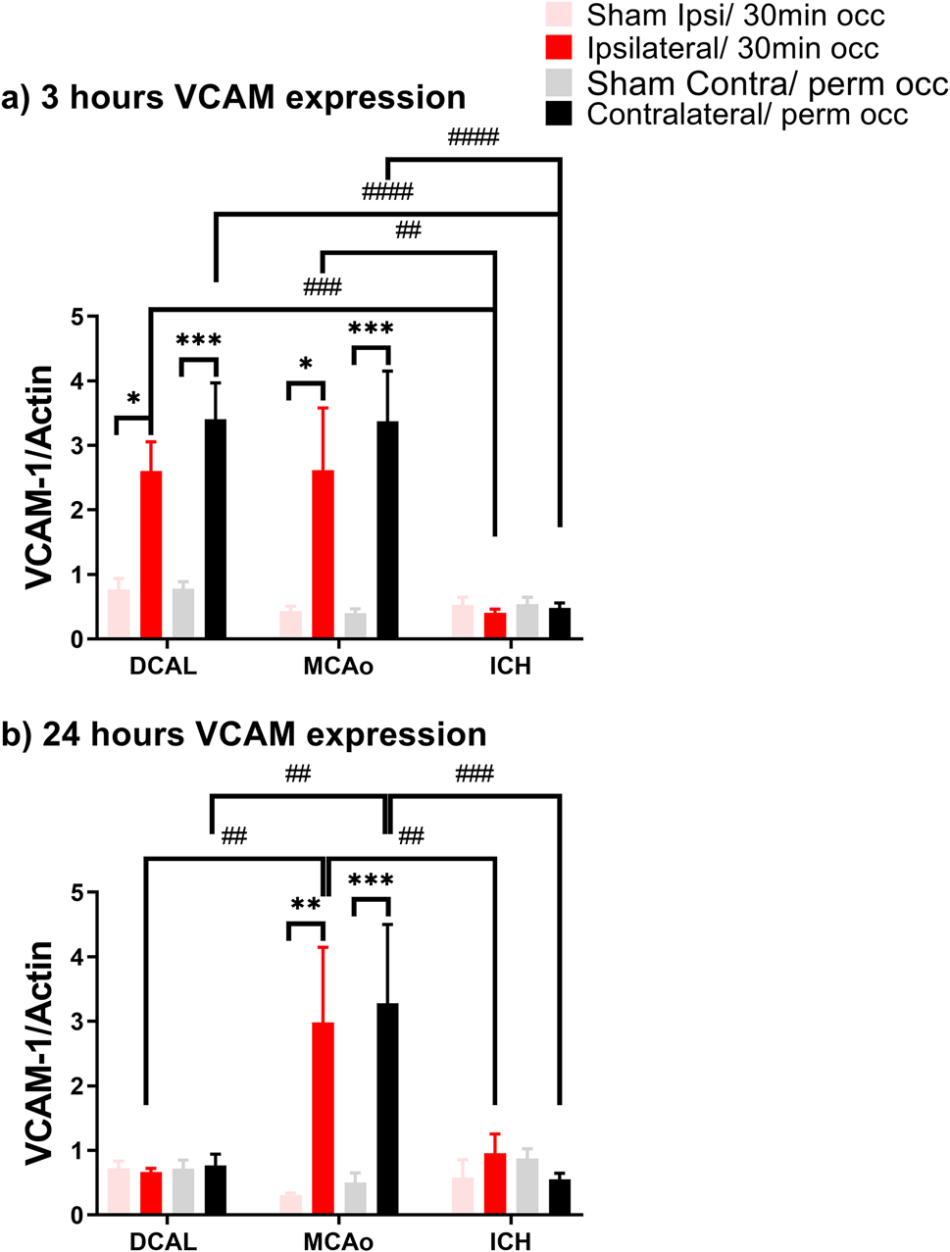
VCAM expression is significantly increased in ischaemic but not haemorrhagic stroke. VCAM expression assessed via western blot shows increased levels in the lesion at (**a**) 3 h post injury only in the DCAL and MCAo model, but at (**b**) 24 h post injury, VCAM expression decreased to baseline levels in the DCAL model, and decreased slightly in the MCAo model. ICH does not result in increased VCAM expression at 3 or 24 h. (Data is Mean ± SEM; Sham: n = 3–5, Stroke: n = 5–10; *p < 0.05, **p < 0.001, ***p < 0.0001, sham vs stroke, ##p < 0.001, ###p < 0.0001, ####p < 0.00001; DCAL vs MCAo vs ICH; two‐way ANOVA with Tukeys post‐hoc analysis).

At 3 h post DCAL-stroke, mice had a 2–threefold upregulation of VCAM in both hemispheres (Fig. 4a) but not at 24 h post injury (Fig. 4b). In the MCAo model, VCAM was significantly upregulated in both sides of the brain at 3 h (Fig. 4a) and interestingly it was still upregulated at 24 h post-MCAo (Fig. 4b). In contrast, in the ICH model, VCAM was not upregulated at either timepoint (Fig. 4a, b).

### Expression of complement receptors and inflammatory cytokines but not chemokines is increased at 24 h post-DCAL

**Figure 5:**
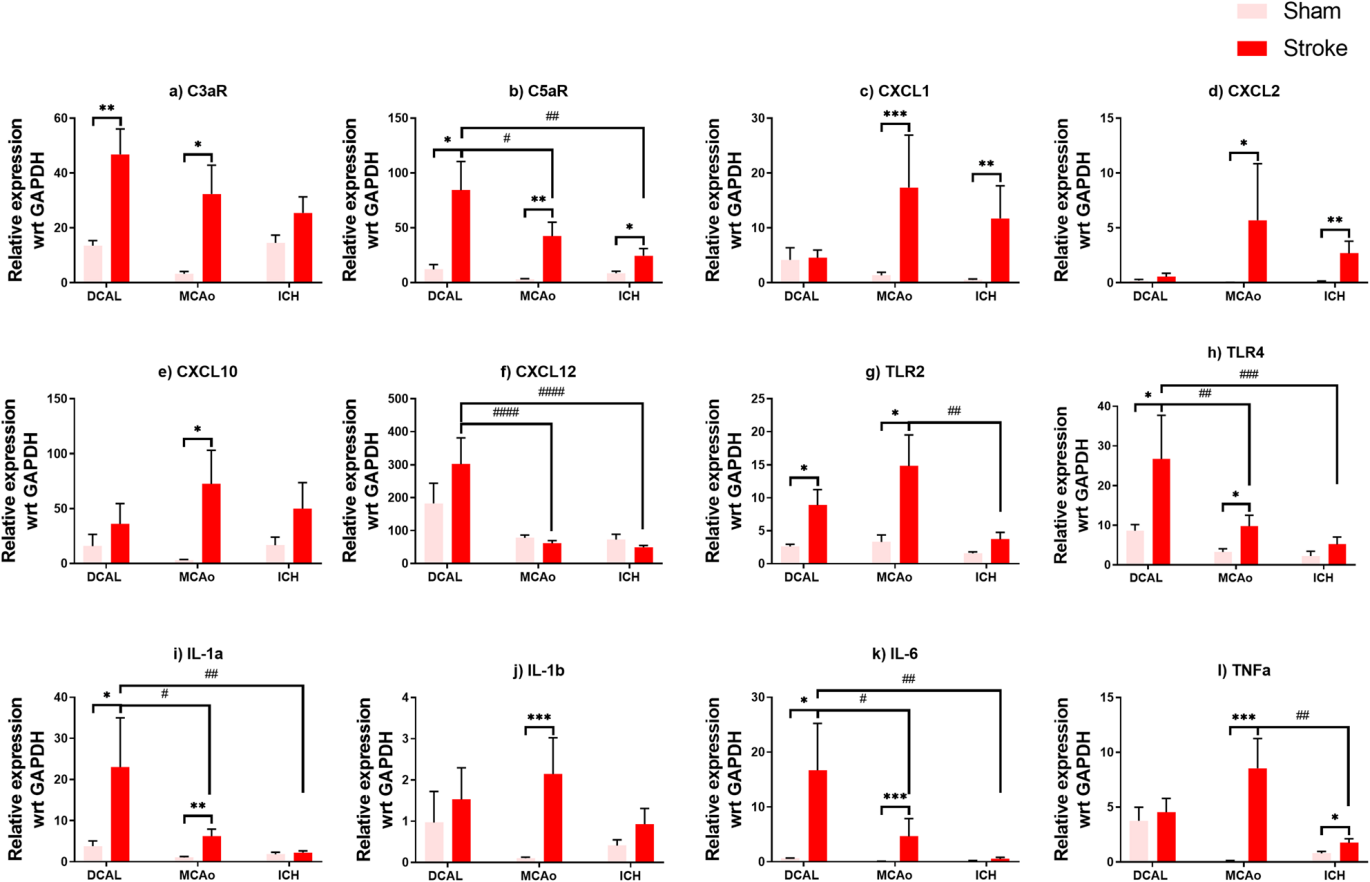
Gene expression analysis of inflammatory genes highlights significant differences between all three stroke models. Real-time PCR analysis of RNA isolated from the site of injury at 24 h shows an overall increase of Complement receptors (**a**) C3aR and (**b**) C5aR expression in DCAL, MCAo and ICH models. There was only a significant increase in chemokines (**c**) CXCL-1 and (**d**) -2 after ICH, and CXCL-1, -2 and (**e**) -10 after MCAo but not in (**f**) CXCL-12. There was an overall increase in (**g-h**) TLR 2/4 gene expression after ischaemic stroke, but there was no discernible increase after haemorrhagic stroke (**i-l**) Inflammatory gene expression was high variable; only MCAo resulted in an upregulation of all the inflammatory genes except IFNγ. (Data is mean ± SEM; Sham: n = 5–9, Stroke: n = 5–8; *p < 0.05, **p < 0.001, ***p < 0.0001; t-test, sham vs stroke, #p < 0.05, ##p < 0.001, ###p < 0.0001, ####p < 0.00001, two‐way ANOVA with Tukey post‐hoc analysis, DCAL vs MCAo vs ICH).

Receptors for complement proteins C3aR and C5aR were upregulated 3 and sevenfold respectively after DCAL (Fig. 5a, b). After MCAo, both C3aR and C5aR were increased tenfold (Fig. 5a, b). However, only C5aR was upregulated in the ICH model (Fig. 5a, b). C5aR is also significantly more upregulated after DCAL stroke unlike MCAo and ICH models.

Expression of CXCL-1, -2, -10 and -12 was not increased after DCAL (Fig. 5c–f) but CXCL-1, -2 and -10 were drastically upregulated after MCAo (Fig. 5c–f). Modest but significant upregulation of CXCL-1 and -2 was seen after ICH (Fig. 5c, d). However, expression of CXCL12 was significantly higher in the DCAL model when compared to MCAo and ICH. Remarkably, because of an evident sham effect in this model, the difference between DCAL and sham was not significant.

There were three- and fivefold increases in expression of TLR2 and TLR4 in the DCAL and MCAo models respectively (Fig. 5g, h) and DCAL stroke had the highest expression of TLR4 within the models. Unlike for ischaemic stroke, there were no significant increases of TLRs in the ICH model (Fig. 5g, h).

Expression of IL-1α and IL-6 significantly increased after DCAL (Fig. 5i–l) and was also the most upregulated in the stroke models. Similarly, in the MCAo model, IL-1α, IL-1β, IL-6 and TNFα increased exponentially while only TNF-α was upregulated at 24 h after ICH (Fig. 5i–l). The inflammatory response in the DCAL-sham animals is once again evident for IL-1β and TNFα (Fig. 5j, l). IFN-γ was not upregulated at this timepoint in any of these models (not shown). HIF1α was only upregulated after ischaemic stroke and significantly upregulated after DCAL stroke when compared to MCAo (Fig. S3).

## Discussion

Here we investigated and compared biochemical and neurobehavioural changes at 24 h in a new model of global forebrain ischaemia with focal stroke, including MCAo and ICH.

Global ischaemia models induce widespread hypoxic-ischaemic injury due to oligemia and I/R injury and recapitulate the neurological effects of cardiac arrest and profound systemic hypotension due to peripheral haemorrhage, strangulation or drowning^8^. The DCAL model is most similar to the transient BCCAo model^9^ and the mouse model of global cerebral ischemia wherein the bilateral common carotid artery occlusion is combined with isoflurane-induced hypotension^10^. Other variations include the bilateral common carotid artery stenosis (BCAS)^11^ and bilateral internal carotid artery occlusion (BICAO) models ^12^.

We showed that in this model blood flow is restricted across the frontal, parietal and temporal lobes of the brain during the ischaemic period. Without occlusion of the basilar artery, the cerebellum remains perfused (not shown) indicating patent and well‐established posterior collateral supply via the Circle of Willis. Therefore it is a model of global forebrain ischaemia. After 30 min when the vascular clip on the right CCA is removed, both sides of the brain reperfuse immediately (Figure S1). Therefore the DCAL model allows us to study the contribution of I/R injury following a transient period of ischaemia as well as provides the means to directly compare the effects of 30 min of global to focal cerebral ischaemia (MCAo model).

It appears that hypoperfusion occurs after permanent suturing of the left CCA for the sham procedure as the extent of Evan’s blue perfusion in these animals is not as high as in naïve untreated animals. Further there are pathological changes, particularly in the inflammatory response, albumin extravasation and endothelial activation in the sham animals that are not seen in the MCAo and ICH sham groups. Indeed, oligemia following CCA ligation is known to cause an inflammatory response without resulting in a quantifiable infarct^13^. Therefore in the DCAL model, we can isolate effects of oligemia and I/R injury from hypoperfusion, although further experiments are needed to confirm this.

Neuronal cell death following global ischaemia occurs by a different mechanism compared to focal ischaemia^14^. At 24 h after DCAL-stroke, mean MRI-based infarct volumes were 15.6 mm^3^ for the left side of the brain (ipsilateral to the site of permanent ligation) and approx. half i.e. 8.8mm^3^ for the right side of the brain (site of transient occlusion). This demonstrates that although reperfusion injury may have been of greater magnitude on the right side of the brain, the left side of the brain suffers the added effect of oligemia which exacerbates cell death that is otherwise not caused by mild hypoperfusion likely seen in the sham cohort. The predominantly striatal lesions are consistent with published global ischaemic brain injury models such as BCCAo^15^. Ischaemia reduces the metabolic rate within the tissue and hence there is more cell loss in the striatum because it is the region of the brain with the highest degree of blood flow-glucose metabolism dissociation^16^. Notably, the overall infarct volume in both hemispheres was still significantly less than that of the unilateral focal infarct caused by MCAo (analysis not shown). Despite this, with a median Bederson score of 4, overall neurological outcome was worse in this cohort when compared to the MCAo cohort. Pronounced neurological deficit after DCAL-stroke was reinforced by the finding of increased footslips/m in this cohort, which was ~ 4.5 × than that observed in the MCAo cohort. However, these neurological impairments did not impact the mobility of these mice or their locomotor activity over a 5 min period. This can be explained by the hyperactive and anxious behaviour observed in these mice. Indeed, an increase in locomotor activity and anxiety has been reported in rodent models of global cerebral ischaemia^17^ as well as in humans^18^, and has been attributed to post-ischaemic microglial activation in the brain^19^.

The intraluminal thread model used to cause MCAo is relatively non-invasive and results in a large infarct localised to striatal regions, particularly basal ganglia and cortex; it is typically considered as a clinically relevant model of large vessel occlusion^20^. At 24 h after 30 min transient MCAo, median Bederson score was 3 and mean lesion volume was 42.3mm^3^, consistent with a recent report that documented Bederson scores of 3–5 and mean lesion volume of 77.5mm^3^ after 45 min of MCAo^21^. Mice subjected to MCAo stroke had quantifiable neurological deficits both in terms of distance travelled and time immobile when compared to the sham operated control mice. Additionally, there was a ~ threefold increase in the number of footslips per metre measured by the ANYMaze system further attesting to the significant neurological dysfunction caused by the MCAo model, and consistent with published reports^22^.

The collagenase injection model is most widely used and best recapitulates blood vessel rupture leading to bleeding, re-bleeding, and consequent haematoma expansion^23^. Although enzymatic disruption of the extracellular matrix is also observed in haemorrhagic infarctions and traumatic haemorrhages in humans, the use of collagenase has its drawbacks. By degrading collagen IV, a part of the extracellular matrix in the brain, collagenase itself affects BBB integrity, alters immune responses as well as causes significant inflammation, which can affect how ICH progresses. The collagenase model is nevertheless suited to this study because it is reproducible, produces a larger injury and causes greater and longer lasting neurological deficits^24^. We found that although the infarct volume was comparable to DCAL stroke, overall neurological dysfunction was less than both ischaemic models. However, similar to focal ischemia, mobility was significantly impaired after ICH.

Following ischaemia, caspase-3 is cleaved and activated and subsequently degrades several substrates in the cytoplasm and nucleus, precipitating in cell death^25^. Our findings are consistent with published data investigating both rat^26^ and mouse^27^ MCAo models; further, activated caspase-3 has also been detected in post-mortem brain tissue from humans who have sustained stroke following occlusion of a major artery^28^. The delayed wave of caspase-3 activity that has been observed at > 12 h post-MCAo defines the secondary expansion of the lesion by energy dependent caspase activation leading to apoptosis and therefore identifies the ischaemic penumbra^29^. After MCAo-stroke we found a 5 × increase in apoptotic activity within the ischaemic lesion. Increased caspase-3 activity is predominately localised to the CA3 region of the hippocampus and striatum 24 h after global ischaemia induced in the BCCAo model^9,30^. We observed that overall, after DCAL-stroke apoptosis in the ischaemic lesion was comparatively less than that of the MCAo cohorts. Caspase3/7 activity doubled in the site ipsilateral to the transient 30 min occlusion but was 3 × in the site ipsilateral to the permanent occlusion, accurately mirroring the infarct volume data. Increased cytochrome c, a pre-cursor to caspase-3 cleavage in the intrinsic apoptosis pathway has been reported at 24 h in rat ICH models involving autologous blood-injection ^31^ and increased apoptotic activity has been reported at the same time point in rat^32^ and mouse models^33^ of collagenase injections. Caspase activity was increased fourfold in the ipsilateral cortex in our model, which was ~ 2 × that seen in the MCAo and DCAL models at the same timepoint. After ICH, added pathophysiological processes such as mechanical damage caused by mass effect of extravasated blood, influx of blood components including complement molecules and coagulation factors, and haemoglobin toxicity^31^ might be promoting apoptosis compared to that seen in ischaemia models.

In the DCAL model, even though infarct volume is less, overall BBB breakdown is worse than in MCAo. While there is a modest increase in the side of the brain ipsilateral to temporary occlusion at 3 and 24 h, there is a significant 2 × increase in albumin extravasation in the side of the brain ipsilateral to the permanent occlusion at 24 h. There is a clear effect of the sham procedure on albumin levels in the DCAL model, demonstrating that hypoperfusion as a result of permanent ligation of the left CCA itself disrupts the neurovascular unit. Presumably, the increase in albumin seen in the side of the permanent occlusion is the net impact of oligemia/hypoxia and I/R injury, whereas that seen in the side of transient occlusion is purely due to I/R injury. BBB disruption is higher due to reperfusion injury in focal transient MCAo models when compared with permanent MCAo. After transient MCAo, there is a biphasic pattern of BBB permeability; there is an increase at 3 h^34^ that is maintained up to 24h^35^, followed by another increase in BBB permeability 48 h after reperfusion^34^. Our data showing increased albumin extravasation 3 h after transient MCAo and persisting for 24 h is consistent with previous publications.

Following haemorrhagic stroke, bleeding in the brain triggers an influx of all blood proteins and therefore measuring BBB permeability is best done by intravenously injecting fluorescent tracers of different molecular weights after ICH and studying how they accumulate in the brain. Using a radiolabelled tracer, Lee and colleagues were able to show increased BBB permeability in rats subjected to intracerebral thrombin infusion^36^. Because our study was designed to compare ischaemic and haemorrhagic stroke, we studied albumin extravasation in all three models. Additionally, for the ICH model, by studying haemoglobin and albumin we were able to perform statistical correlation studies to demonstrate that at 24 h post-ICH there is a significant increase in BBB permeability in the peri-haematomal region. It is well known that BBB disruption after ICH is caused by the release of iron (due to breakdown of haemoglobin) after red blood cell lysis and subsequently results in formation of vasogenic oedema^37^.

Cerebral microvascular endothelial cells are rapidly activated after a stroke and upregulate a range of pro-inflammatory factors, including VCAM-1. VCAM-1 promotes adhesion and migration of peripheral leukocytes and BBB damage. Gauberti et al. conducted a thorough analysis of VCAM-1 using MR-imaging of microparticles of iron oxide (MPIOs) targeted to VCAM-1 via an antibody. They demonstrated that VCAM-1 is globally upregulated within 24 h after MCAo and persists for at least 5 days, thereby providing evidence of an inflammatory penumbra in this model^13^. Our findings confirm that VCAM-1 is indeed upregulated both in the ipsilateral and contralateral sides of the brain at 24 h post-MCAo. Gauberti et al. also confirmed enhanced VCAM-1 upregulation after ischaemia/oligemia^13^; while our data show that VCAM-1 is upregulated in both sides of the brain in the DCAL model, and this is over and above that caused by oligemia likely seen in the sham cohort. Others have also confirmed VCAM-1 upregulation in global ischaemic brain injury^38^. Taken together, VCAM-1 upregulation seen at 3 h but not at 24 h after bilateral forebrain ischaemia via the DCAL model can be attributed predominantly to I/R injury.

However, our findings that VCAM-1 is not upregulated at 3 or 24 h in the haematomal or perihaematomal (Fig. 4a, b) region after ICH do not align with the haemorrhagic stroke data from Gauberti et al.^13^ which showed a wide perihaematomal distribution of VCAM-1 in the acute phase of ICH in the same model as ours. This could be due to the high sensitivity of specific molecular localisation of VCAM and high resolution imaging in their study when compared to conventional immunoblotting methods that we utilised. Also, the dose of collagenase was 3 × more concentrated what we used, and hence corresponds to a more severe injury. Consistent with our findings, Liesz et al. also showed that VCAM-1 is not upregulated until 72 h post-ICH and only in the autologous blood injection model not the collagenase model^39^.

During ischaemic injury to the brain, free radical release activates the pro-inflammatory nuclear transcription factor NF-κB which triggers the transcription of genes encoding pro-inflammatory cytokines IL-1 α and β, IL-6, TNF-α. TNF-α and IL-1α in turn upregulate endothelial bound VCAM-1^16^. In addition to an increase in VCAM-1 mRNA (not shown), we found a significant increase in transcription of these cytokines within the lesion, 24 h after MCAo, consistent with published reports^40^. In contrast only IL-1 α and IL-6 were upregulated in the side of the brain corresponding to the transient occlusion at this timepoint after DCAL stroke. Although TNF-α is known to be upregulated in global brain ischaemia, it has a biphasic response peaking at 3 h and then again at 36 h^14^. In our data, it is interesting that there is lack of a significant change in TNF-α mRNA levels after the DCAL procedure primarily because of the sham effect wherein TNF-α levels in the brains of sham animals are markedly higher than that seen in the sham controls from the MCAo cohort. Similar to the albumin data, this further supports the pronounced inflammatory effect of oligemia and demonstrates that I/R injury does not trigger an additional increase in TNF-α mRNA at this timepoint. It remains to be determined whether there is an early increase in TNF-α corresponding to the VCAM-1 upregulation at 3 h post-DCAL as it is a known inducer of VCAM-1^40^. In contrast, after haemorrhagic stroke, only TNF-α was significantly upregulated at 24 h. In both the collagenase and autologous blood injection models of ICH, microglial produced IL-1, TNF-α and IL-6 peak early at 3 h, while IFN-γ peaks only at 7 days and coincides with the delayed invasion of lymphocytes after ICH^39^.

Chemokines can be secreted by a range of cell types following stroke^41^. Following focal ischaemia increased chemokine expression promotes leukocyte infiltration and rapid peri-infarct upregulation of CXCL1 and CXCL10 occurs has been observed in a rodent model of permanent ischaemia^42^. We found that CXCL-1, -2 and -10 were all upregulated after focal ischaemia but not in global forebrain ischaemia induced by the DCAL model. In contrast, CXCL12 and HIF-1α were highly upregulated only after DCAL albeit there is a strong sham effect for CXCL12. HIF-1α is a transcription factor commonly upregulated during cerebral ischaemia to help neuronal cells adapt to the lack of oxygen^43^. Similar to other models of carotid occlusion^30^, and consistent with the neural response on hypoxia, in the DCAL model we saw significant upregulation of HIF-1α. Also known as SDF-1 (stromal-derived factor-1), CXCL12 expression is known to be regulated by the HIF-1 α in endothelial cells in response to reduced oxygen tension^43^. This could explain why both HIF-1 α and CXCL12 are concomitantly upregulated in the mouse brain after DCAL. The increase in expression of CXCL12 in the sham animals further suggests that there is an effect of hypoxia due to permanent ligation of the left CCA in these animals.

Following ICH, activated microglia release chemokines that promote recruitment of peripheral inflammatory cells and produce pro-inflammatory cytokines^44^. We found that expression of CXCL1 and CXCL2 was significantly elevated at 24 h post-ICH, potentially secreted by astrocytes and mediated via TLR2 which is induced at 6 h post-collagenase infusion^45^. However, we did not detect a significant increase in TLR2 expression at this timepoint and it remains to be determined whether TLR2 is upregulated at 6 h in our model. Neither did we find a significant change in TLR4 expression at this timepoint, unlike published reports showing significant upregulation of TLR4 in peri-haematomal tissue in the autologous blood injection model at the same timepoint^46^. In contrast, both TLR2 and TLR4 were significantly increased after focal and global ischaemia. During ischaemic injury, microglial TLR2 stimulation results in increases in TNF-α, IL-6 and IL-10, and TLR4 stimulation results in increases in TNF-α, IL-6, IL-10, CXCL-10 and IFN-β^47^. These data reveal the similarities in the molecular response to I/R injury regardless of the animal model chosen.

There is cumulative evidence that activation of complement anaphylatoxins is a key pathogenic mechanism of ischaemic brain injury. Under resting conditions in the brain, receptors for the complement C3a and C5a are predominately expressed by neurons, but under inflammatory conditions both glial cells and neurons express C3aR and C5aR. However, following focal ischaemic brain injury, C3aR and C5aR expression on endothelial cells and astrocytes was upregulated from 6–48 h, which promoted neutrophil recruitment and worsened brain injury^48^. C3aR upregulated under conditions of hypoxic ischaemic brain injury in neonatal rodents is conversely linked to improved outcome^49^. Hence although we show that C3aR expression is upregulated in both MCAo and DCAL models, we cannot ascertain if it is protective or detrimental without further studies. After ICH, thrombin activates the complement system to facilitate red blood cell lysis and C3aR has been linked to inflammatory cell infiltration in the autologous blood injection model of ICH^50^. We found a significant upregulation of expression of C5aR and not C3aR in our ICH model, at 24 h post-haemorrhage. C5aR activation is also associated with worsened outcome after ICH, and mice treated with C5a receptor antagonist showed less granulocyte infiltration into the lesion^51^.

In Table 1 we have summarised our findings about the DCAL model and how it compares to the MCAo and ICH models. It should be noted that we studied male C57Bl6 mice only and we will need further studies to confirm if the same trends will be obtained in female mice and other strains of mice.

**Table 1:** Comparison of secondary brain injury in the DCAL, MCAo and ICH models.

	DCAL	MCAo	ICH
**Neurological dysfunction **(**relative to sham**)			
Median Bederson Score	4	3	2
Footslips/m	Fourfold increase	Two fold increase	Two fold increase
Distance travelled	No significant decrease	50% decrease	30% decrease
Time mobile	No significant decrease	40% decrease	35% decrease
**Infarct volume**	Average volume 10 mm^3^ on both hemispheres	Fourfold larger compared to DCAL and ICH	Average volume 12 mm^3^ on ipsilateral hemisphere
**Apoptosis** (Caspase-3 activity)	Significant increase in both hemispheres	Five fold ↑	Four fold ↑ -Highest apoptotic activity
**Blood–brain barrier damage** (albumin extravasation)	Only significantly increased (2.5 fold) at 24 h on side of permanent occlusion	Two fold ↑ in ipsilateral side at both 3 and 24 h	Four–five fold ↑ in ipsilateral side at both 3 and 24 h -Highest albumin levels
**VCAM expression**	Threefold increase at 3 h on side of 30 min occlusion	Eight–ten fold increase in both hemispheres at both 3 and 24 h -Highest VCAM expression	Was not increased on either side at 3 h and 24 h
**Gene expression**			
Complement	↑ C3aR and C5aR	↑ C3aR and C5aR	↑ C5aR
Chemokines	No significant upregulation	↑ CXCL-1, -2 and -10	↑ CXCL-1, -2
TLRs	Two–fourfold ↑ TLR2 and TLR4	Three–fivefold ↑ TLR2 and TLR4	No significant upregulation
Inflammation	↑ IL-1α and IL-6	↑ IL-1α, IL-1β, IL-6 and TNFα	↑ TNFα

## Conclusions:

The initial triggers are different for ICH, ischaemic stroke and global ischaemic brain injury, and the reason for ensuing brain injury differs in each type of stroke. But as us and others have shown, all types of stroke have a final common pathway- cell death, endothelial activation contributing to arterial wall damage, inflammation and BBB breakdown. Certain interventions such as blood pressure lowering drugs have already been trialled in cases of ischaemic and haemorrhagic strokes with variable outcomes^52^. Anti-inflammatory or endothelial protective drugs are also likely to be protective for both ischaemic and haemorrhagic stroke. Patients with ischaemic cerebrovascular disease have a high risk of ICH, and vice versa. Thrombolysis after ischaemic stroke is also associated with increased risk of ICH^53^. For these reasons we established and characterised uniform readouts across rodent ischaemic and haemorrhagic stroke models. We found that although cell death was comparable between the global and focal ischaemia models, neurological outcome and albumin extravasation were significantly worse in the site of permanent occlusion after global forebrain stroke. Molecular responses including VCAM-1 upregulation and transcriptional upregulation of pro-inflammatory cytokines and TLRs/complement differed vastly in the three models. These techniques and models can help develop new therapies for I/R injury that can be tested for safety in models of haemorrhagic stroke. They can also further aid in the pre-clinical development of pharmacotherapies that are safe for all stroke types and therefore preclude need for neuroimaging prior to administration.

## Experimental procedures

### In vivo experiments

#### Mice

All experiments were approved by Alfred Research Alliance Ethics Committee (ethics applications E/1683/16/M, E/1851/18/M and E/1937/19/M) in accordance with Australian code for the care and use of animals for scientific purposes 8th Ed 2013 and in compliance with ARRIVE guidelines for reporting animal experiments. Refer to supplementary methods for further information.

#### MCAo

Transient focal cerebral ischaemia (30 min) was induced using MCAo, as described previously^54^. Briefly, mice were anesthetized with isoflurane (1.5–2%), and rectal temperature was maintained at 37 °C. MCAo was induced by transecting the ECA, temporarily tying off the CCA and using the external carotid artery trunk as a side path to pass a suture through the internal carotid artery (ICA) to lodge in the junction of the anterior and middle cerebral arteries. A silicone rubber-coated monofilament suture was inserted via the right ECA until it obstructed the MCA, and the common carotid artery was allowed to perfuse for the duration of the ischemic period of 30 min. CBF was measured using transcranial laser Doppler flowmetry (Periflux System 5010; Perimed) in the centre (bregma coordinates: 2 mm posterior, 5 mm lateral) of the ischemic territory. For sham controls, vessels were visualized and cleared of connective tissue under anaesthesia. All animals were left to recover on the heat pad for at least 4 h after procedure.

#### Double carotid artery ligation (DCAL)

Transient global forebrain ischaemia was induced by permanently ligating the left common carotid artery (CCA), and transiently clamping the right CCA. To permanently occlude the left CCA, fat and the surrounding connective tissue was dissected and a 2–3 cm length of 5–0 silk suture was tied around it, taking care not to include the nerve. After a 10 min rest period to minimise overstimulation of the vagal nerve, the right CCA was cleaned of fat and connective tissue, and a vascular clip was used to transiently clamp the artery for 30 min after which, it was removed and the animal was recovered for 24 h. This model was specifically designed to assess I/R injury independently of the effect of ischaemia/oligemia and therefore, for sham controls, the left CCA was permanently ligated, and the mice were allowed to recover. There were no deaths as an outcome of this procedure.

To determine the extent of ischaemia and reperfusion following DCAL, mice were subjected to DCAL procedure; in one cohort both arteries were ligated and in the other the right common carotid artery was transiently occluded as is done in the DCAL procedure. Sham procedure whereby the left CCA was permanently occluded, and naïve animals were also included. Evan’s blue was then intravenously injected and allowed to circulate before mice were culled (Refer to supplementary methods for more information).

#### ICH

Mice were anaesthetised with 2,2-tribromoethanol (Avertin) and 0.15U of Collagenase VII (from Clostridium *histolyticum*; both from Sigma Aldrich Australia) was injected intracerebrally as described^24^ at coordinates MV: 1.75, AP: 0.4, DV: 3.8 from bregma. For sham controls, 1 µl saline was injected at the same location, instead of collagenase. The syringe was held in place for 5 min and gently withdrawn, the hole in the skull was sealed with bone wax, and the animals were left to recover overnight with supportive care as above.

#### Euthanasia and tissue harvesting

Supplementary methods.

#### Behavioural testing

Behaviour was assessed 20–24 h post-stroke as detailed below.

- **Bederson scoring:** conducted as described^55^.
- **Open field and parallel rod floor assays:** Mice were placed in a 30 × 30 cm black Plexiglas box or in a box containing narrow wires 1 cm apart as described^22^. Behavioural activity was tracked using AnyMaze software (Stoelting Co., USA).

#### Magnetic resonance imaging

At 24 h post-stroke, ex-vivo MRI was carried out as described previously^56^. Analysis was performed using ITK-SNAP^57^. Refer to supplementary methods for further information.

#### Cresyl violet staining

After ex vivo MRI, brains were cleaned of agar, embedded in paraffin and 5 µm sections were stained with Cresyl violet at the Monash Histology Platform.

#### Caspase-3/7 activity assay

The fluorogenic substrate (Ac-DEVD-AFC) was used, and the protocol was according to Notte et al^58^.

#### Western Blot

VCAM and β-actin expression was detected with rabbit monoclonal anti-VCAM-1 (1:1000; 0.437 ng/ml; Abcam, Australia) and by goat polyclonal anti-β Actin (I-19)-HRP (1:500; 100 ng/ml; Santa Cruz Australia) respectively.

#### Albumin ELISA

Albumin content in the brain was determined using the Mouse Albumin ELISA Quantitation Set (Bethyl Laboratories, USA) according to the manufacturer’s instructions and as described^59^.

#### Real-time (RT)-PCR

Changes in expression of GAPDH, 18S RNA, C3aR, C5aR, CXCL1, CXCL2, CXCL10, CXCL12, TLR2, TLR4, IL-1α, IL-1β, IL-6, TNFα, IFNγ, HIF1α were assessed using TaqMan RT-PCR (Thermo Fisher Scientific, Australia). Details included in supplementary methods.

#### Statistical analysis

Statistical analysis was performed using Prism 8 software (GraphPad, US). Comparison of experimental datasets was performed by one-way ANOVA with Tukey’s post-hoc correction or two-way ANOVA with Tukey’s or Dunnetts post-hoc correction as stated. Differences between two groups were assessed by two-tailed student t-tests (unpaired or unpaired with Welch’s correction for parametric data and Mann–Whitney test for non-parametric data). P < 0.05 was considered significant.

## Supplementary information

Supplementary Information.
