# Supplementary material for: Characterization of a novel model of global forebrain ischaemia–reperfusion injury in mice and comparison with focal ischaemic and haemorrhagic stroke
Source: Sci Rep. 2020 Oct 23;10:18170. doi: 10.1038/s41598-020-75034-4 (PMC7585423; doi:10.1038/s41598-020-75034-4)
Supplement: Supplementary file 1 — Supplementary Information. [file 41598_2020_75034_MOESM1_ESM.docx]

**SUPPLEMENTAL MATERIAL**

**Title: Characterization of a Novel Model of Global Forebrain Ischaemia-Reperfusion Injury in Mice and Comparison with Focal Ischaemic and Haemorrhagic Stroke**

**Author Names and Affiliations:** Natasha Ting Lee^1^, Carly Selan^1^, Joanne S Chia^1^, Sharelle A Sturgeon ^1^, David K Wright^2^, Akram Zamani^2^, Melrine Pereira^1^, Harshal H Nandurkar^1^**^*^**and Maithili Sashindranath^1^**^*^**

^1^Australian Centre for Blood Diseases, Central Clinical School, Monash University, Alfred Hospital, Melbourne VIC 3004, Australia.

^2^ Department of Neuroscience, Central Clinical School, Monash University, Melbourne, VIC, 3004, Australia

**^*^ These authors contributed equally to this work**

**Running title:** Focal and global ischemia and hemorrhagic stroke

**Corresponding author:** Dr Maithili Sashindranath, Australian Centre for Blood Diseases, Central Clinical School, Monash University

**Present address:** Monash AMREP building, Level 1, Walkway, via The Alfred Centre, 99 Commercial Road, Melbourne, VIC 3004, Australia.

**Email:** maithili.sashindranath@monash.edu

**Phone:** +61 3 9903 0155

**Fax:** +61 3 9903 0228

**Supplemental Methods**

**Animals:** Experiments were performed on male C57BL/6 mice (age, 8 ± 0.2 weeks; weight, 28.2 ± 2 g) obtained from an in-house colony. Only male mice were included as stroke affects different sexes differently. Mice were randomly assigned to the various experimental groups, and provided with food and water ad libitum. Each mouse was assigned a unique serial number identifier and all neurological and biochemical tests were performed and analysed in serial order with the groups blinded to the assessor. Animals were left to recover on a heat pad for at least 4 hours after procedures and were monitored every 20 minutes during that period. Thereafter, mice were housed individually and given supportive treatment including warmth and mashed food. They were monitored hourly again from 8 am the next day until the time of culling. Because we were comparing stroke and sham groups and not effects of drug treatment, formal size assessment was not conducted. It should be noted that we studied male mice only and we will need further studies to confirm if the same trends will be obtained in female mice.

**MCAo model:** All animals were given buprenorphine (0.05 mg/kg) subcutaneously prior to the surgery and after as needed. 6 mice out of 60 suffered a subarachnoid haemorrhage, which is a common outcome of MCAo, and was excluded from the study.

**ICH Model:** Animals were given buprenorphine (0.05 mg/kg) subcutaneously prior to surgery and 6h later. Two animals succumbed to the procedure overnight.

**MRI protocol:** Imaging was performed at the Alfred Research Alliance- Monash Biomedical Imaging (ARA-MBI) Pre-clinical Imaging Facility with a 9.4 T/20 cm Bruker MRI with actively decoupled volume transmit and surface-receive coils. Diffusion-weighted images were subsequently averaged and the infarct was traced using ITK-SNAP (www.itksnap.org) by an investigator blinded to the experimental condition. Infarct volumes were calculated using fslstats, part of the FMRIB Software Library (FSL). A 3D diffusion-weighted image was acquired with a 2-shot echo planar sequence and the following imaging parameters: repetition time/echo time = 5,000/33 ms; field of view = 19.2 x 19.2 x 7.6 mm^3^; matrix size = 96 x 96 x 38; and resolution = 200 x 200 x 200 μm^3^. In addition to a single non-diffusion (b0) image, diffusion weighting was applied in 81 directions with diffusion duration = 5 ms; diffusion separation = 12 ms; and b-value = 4,000 s/mm^2^.

(3) Evan’s blue injection to assess the extent of ischaemia and reperfusion following DCAL

To visualise and quantitate the extent of ischaemia and subsequent reperfusion of the brain during DCAL, Evans blue was used. Mice were subjected to DCAL procedure and in one cohort both arteries were ligated and in the other the right common carotid artery was transiently occluded as is done in the DCAL procedure. Evan’s blue (0.2 g/kg; Sigma–Aldrich Australia) was injected intravenously soon after. After 3-5 minutes of circulation, the mice were culled and the brain was harvested. By intravenously injecting Evans Blue, the parts of the brain that are devoid of blood flow during the DCAL procedure would not be stained blue. It would also demonstrate how effective reperfusion of the vessel is after the transient occlusion when the vascular clip is removed following 30min of DCAL.

As illustrated in Fig S3 C, the brain was sectioned in 2mm slices using the mouse brain blocker, spanning the region of bregma +2mm anterior and 0-4mm posterior as per the mouse brain atlas (<http://labs.gaidi.ca/mouse-brain-atlas/>) using the Mouse Brain Blocker (David Kopf Instruments, USA), and imaged on the Odyssey CLx imager (Li-COR, USA; not shown). The sections were then divided into the left and right hemispheres and homogenised 300mg wet weight of tissue per 1ml of Lysis Buffer (TBS +1% Triton X-100). For quantitation purposes, the lysates were transferred to a 96 well microplate and read again on the Odyssey CLx imager. Brain lysates obtained from an untreated mouse brain was used as a blank control. Data was depicted as OD_700nm_- blank.

**Western Blot:** Whole brain lysates were prepared from mice subjected to MCAo, DCAL and ICH procedures and killed at 3 and 24h. Lysates were snap frozen on dry ice and kept at -80^o^C until use. 20μg of total protein was subjected to SDS-PAGE (10% gel electrophoresed at 250V for 1h). VCAM and Actin levels were detected by rabbit monoclonal anti-VCAM-1 (1:1000; 0.437ng/ml; Abcam, Australia) and by goat polyclonal anti-Actin (I-19)-HRP (1:500; 100 ng/ml; Santa Cruz Australia) respectively.

**Haemoglobin Assay:** Haemoglobin levels in the brain were determined using the Haemoglobin Assay Kit (Abnova, Taiwan) according to the manufacturer’s instructions.

**Real-Time (RT)-PCR:** Sections of ischemic/haemorrhagic brain tissue after MCAo, DCAL, and ICH were dissected and kept in RNAlater (Thermo Fisher Scientific, Australia) for 24 hours before being stored at -80°c until use. For the DCAL model, the site corresponding to the transient occlusion was dissected. For RNA extraction, tissues were homogenized in RLT lysis buffer (Qiagen, Hilden, Germany) and DNA-free RNA was prepared using RNeasy Mini Kit and DNase I set (Qiagen, Hilden, Germany) according to manufacturer’s protocols. Identical amounts of total RNA (1µg or 0.8µg per sample) were used to synthesise complementary DNA through reverse transcription reaction and real-time PCR was performed as previously described^34^. Genes that were studied are as described below, with gene expression assays purchased from Thermo Fisher Scientific, Australia.

| Gene Name | Gene Expression Assay |
| --- | --- |
| GAPDH | Mm99999915_g1 |
| 18S | Mm04277571_s1 |
| C3aR | Mm02620006_s1 |
| C5aR | Mm00500292_s1 |
| CXCL1 | Mm04207460_m1 |
| CXCL2 | Mm00436450_m1 |
| CXCL10 | Mm00445235_m1 |
| CXCL12 | Mm00445553_m1 |
| TLR2 | Mm00442346_m1 |
| TLR4 | Mm00445273_m1 |
| IL-1α | Mm00439620_m1 |
| IL-1β | Mm00434228_m1 |
| IL-6 | Mm00446190_m1 |
| TNFα | Mm00443258_m1 |
| IFNƔ | Mm01168134_m1 |
| HIF1α | Mm00468869_m1 |

**Euthanasia and tissue harvesting**: At 24 hours post-surgery, mice were anaesthetized with ketamine/xylazine (K/X; 85 mg/kg; Ketamav 100, MavLab, Australia/15 mg/kg i.p.; Ilium Xylazil‐20, Troy Laboratories, Australia), and transcardially perfused with phosphate buffered saline (PBS) pH 7.4. Unless otherwise stated, the infarcted portion of the brains were dissected and homogenized to 300mg wet weight of tissue per 1ml of Lysis Buffer (TBS +1% Triton X-100 + 1% protease inhibitors; Roche Australia).

**Supplemental Results**

(1) Extent of ischaemia and reperfusion following DCAL assessed via Evan’s blue injection

**Supplementary Figure 1:** (A) On the side of permanent occlusion, Evan’s Blue at t=0 signal was significantly increased in the section 2mm anterior to bregma, after reperfusion in the DCAL model as compared to mice in which bilateral common carotid artery occlusion was maintained (DCAL non-perfused). The amount of Evan’s blue in the non-perfused brain was also significantly different to mice subjected to sham procedure at t=0 as well as in naïve untreated mice. In the mid-section, (0-2mm posterior to bregma) only the naïve animals showed significantly increased Evan’s blue accumulation compared to the non-perfused DCAL mouse brains (B) On the side of transient occlusion, Evan’s blue signal was most significantly increased in the middle section (0 to 2mm posterior to bregma) (Data is Mean ± SEM; n=3; *p<0.05, **p<0.001, ****p<0.00001; repeated measures two-way ANOVA with Tukeys post hoc analysis). (C) Schematic depicting the coordinates of the mouse brain sections analysed in A and B. (Ctx= cortex, Hipp= hippocampus, Str= striatum, Th= thalamus, Hypo= hypothalamus).

1. Haemoglobin levels are increased at 3 hours and 24 hours post-ICH

Haemoglobin levels were measured to determine the amount of blood in the brain as a result of ICH. As expected, there was a 400% increase in haemoglobin levels in the ipsilateral side 3 hours post ICH (n=5-8, p=0.0001) (Figure S2a). At 24 hours post injury, there was still significant amount of haemoglobin in the ipsilateral side of the brain (n=5-8, p=0.0053) (Figure S2b).

**Supplementary Figure 2: After ICH, haemoglobin levels within the brain tissue are significantly increased** at both **(a)** 3 hours and **(b)** 24 hours (Data is Mean ± SEM; Sham: n=5-6, stroke: n=7-9; **p<0.001, ****p<0.00001; two‐way ANOVA with Tukey post‐hoc analysis.)

HIF-1α expression is increased at 24 hours post-ischaemic but not haemorrhagic stroke

1. Expression of HIF-1α was increased after DCAL and MCAo but not after ICH

**Supplementary Figure 3**: Significant upregulation of HIF-1α gene expression after DCAL stroke, and lower yet significant upregulation after MCAo. Interestingly, there was a slight downregulation after ICH. (Data is Mean ± SEM; Sham: n=5-9, Stroke: n=5-8; *p<0.05; unpaired t-test; stroke vs. sham; #= p<0.05; DCAL vs MCAo, ICH)
